# Aluminium Accumulation and Intra-Tree Distribution Patterns in Three *Arbor aluminosa* (*Symplocos*) Species from Central Sulawesi

**DOI:** 10.1371/journal.pone.0149078

**Published:** 2016-02-12

**Authors:** Marco Schmitt, Sven Boras, Aiyen Tjoa, Toshihiro Watanabe, Steven Jansen

**Affiliations:** 1 Institute of Systematic Botany and Ecology, Ulm University, Ulm, Germany; 2 Agriculture Faculty, Tadulako University, Palu, Indonesia; 3 Research Faculty of Agriculture, Hokkaido University, Sapporo, Japan; Institute for Sustainable Plant Protection, C.N.R., ITALY

## Abstract

Accumulation of Aluminium (Al) at concentrations far above 1,000 mg kg^-1^ in aboveground plant tissues of *Arbor aluminosa* (*Symplocos*) species is the main reason why traditional Indonesian weavers rely on their leaves and bark as a mordant for dyeing textile. Recently, *Symplocos* species have become a flagship species for the conservation efforts of weaving communities due to their traditionally non-sustainable sampling and increasing demand for *Symplocos* plant material. Here we investigated *Symplocos odoratissima*, *S*. *ophirensis* and *S*. *ambangensis* at three montane rainforest sites in Central Sulawesi to measure Al levels in different tissues and organs. The highest Al concentrations were found in old leaves (24,180 ± 7,236 mg·kg^-1^ dry weight, mean ± SD), while young leaves had significantly lower Al levels (20,708 ± 7,025 mg·kg^-1^). Al accumulation was also lower in bark and wood tissue of the trunk (17,231 ± 8,356 mg·kg^-1^ and 5,181 ± 2,032 mg·kg^-1^, respectively). Two Al excluding species (*Syzigium* sp. and *Lithocarpus* sp.) contained only high Al levels in their roots. Moreover, no difference was found in soil pH (4.7 ± 0.61) and nutrient (K, Ca, Fe, Mg) availability at different soil levels and within or outside the crown of Symplocos trees, except for the upper soil layer. Furthermore, a positive and significant correlation between Al and Ca concentrations was found at the whole plant level for *Symplocos*, and at the leaf level for *S*. *ophirensis* and *S*. *ambangensis*, suggesting a potential role of Ca in Al uptake and/or detoxification within the plant. Our results provide evidence for strong Al accumulation in *Symplocos* species and illustrate that both Al accumulation and exclusion represent two co-occurring strategies of montane rainforest plants for dealing with Al toxicity. Indonesian weavers should be encouraged to harvest old leaves, which have the most efficient mordant capacity due to high Al concentrations.

## Introduction

One of the first observations of plants containing high concentrations of alum may have been made by Georg Eberhard Rumphius, when he described the *Arbor aluminosa* (“aluminium tree”) [1]. This tree was later identified as a species of *Symplocos* (Symplocaceae) [2], which includes ca. 250 species native to Asia, Australia, and the Americas. Although aluminium (Al) had not been discovered yet during the time of Rumphius, the notion of high levels of alum in plants was based on the traditional use of plant material as a mordant for dyeing textile, a process that apparently also involved alum in India and South-East Asia [1,3–7]. The first chemical identification of Al salts in leaves of *Symplocos* was made by Driessen [8], and we refer to Hutchinson [9] for a historical overview.

Nowadays, traditional weaving communities across the Indonesian archipelago still rely on the supply of plant material, which includes several *Symplocos* species as a natural mordant and additional plants (e.g., *Morinda citrifolia* and *Indigofera*) for dyes and tannins [10–12]. Although Al accumulation is found in a total of ca. 45 angiosperm families [5,6,13], the dyeing protocol of Indonesian weavers is based on the exclusive use of local species [12]. *Symplocos* species in Indonesia are found in remnant forests and communal forests on deeply weathered soils, but are becoming rare in various areas and hardly fulfil the increasing demand by Indonesian weavers. In fact, *Symplocos* has become a flagship species for the conservation efforts of ca. 13,000 women still working with traditional, natural dyes [12]. Since the traditional harvesting method for obtaining *Symplocos* plant material involves the collection of leaf and bark tissue from living forest trees, which commonly involves the cutting of entire trees, this destructive method frequently results in tree dieback. Moreover, cultivation of local *Symplocos* species has not been prompted yet, and efforts to promote forest sustainability currently focus on the collection of old, fallen leaves from the forest floor [12]. We lack, however, scientific evidence that old leaves contain the highest Al levels, which would also indicate that old leaves have the highest mordant capacity. As far as we know, the highest concentration of Al in aboveground plant tissue measured was 72,240 mg kg^-1^ in *S*. *spicata* Roxb. [4]. Modern chemical analyses of Al concentrations in *Symplocos* plant tissue, however, are limited and include only few tropical species from their natural environment [14–16].

Although Al is the most abundant metal in the earth´s crust and heavily exploited by human activities [17], its availability increases exponentially in acidic soils because a low pH (< 5) changes Al from a crystalline structure into plant available forms, some of which are known to be phytotoxic [18–20]. In fact, Al toxicity represents one of the most limiting factors for crop production in acidic soils [21]. While considerable research has been devoted on Al toxicity of crops, our understanding of Al accumulation in flowering plants is limited to a few Al accumulating species such as *Camellia sinensis*, *Fagopyrum esculentum*, *Hydrangea macrophylla*, and *Melastoma malabathricum* [22–25]. The ecological and evolutionary aspects of how tropical plant species deal with Al toxicity remain poorly studied [13,15,26].

Two main strategies are distinguished with respect to Al^3+^ response mechanisms, namely exclusion and accumulation, which have also been applied to a wide range of other metals [27]. Al exclusion is considered to rely on an external (i.e., apoplastic) resistance mechanism, resulting in Al levels in aboveground tissue that are on average 200 mg kg^-1^, whereas accumulation involves detoxification via an internal (i.e., symplastic) mechanism [13,28–30]. Al accumulators are typically defined as plants that show Al levels in aboveground tissue above 1,000 mg kg^-1^ [13]. How exactly Al is detoxified in aboveground plant tissues and in which chemical forms Al is present remains largely unclear [31]. There is some evidence suggesting that Al can bind to oxalate in the vacuoles of leaf cells and to citrate when being transported through the xylem [22,32–34]. However, it is also clear that detoxification mechanisms can be taxon-specific [16]. Moreover, both the xylem loading, potential transport via phloem, and the ligand exchange are not fully understood [35,36].

It has been suggested that obligate Al accumulators, which typically show a beneficial effect of Al on plant growth [24,37,38], may show higher activity of antioxidant enzymes, reduction of iron toxicity, or other unknown metabolic advantages under acidic soils [39,40]. In a cerrado sensu strictu physiognomy, for instance, only five of the 38 tree species appeared to be Al accumulators, although these accounted for 30% of the total importance value in the community [41]. Also, the spatial distribution of Al accumulating trees in West Sumatra showed a positive correlation with the soil edaphic status [42]. These findings may suggest that there could be potential trade-offs in the costs and benefits of Al exclusion and accumulation that may provide evolutionary fitness advantages [43]. It has also been suggested that Al accumulation may provide a defence mechanism against herbivory or pathogens [31], similar to metals such as nickel (Ni) and selenium (Se) [44,45].

In this study, we aim to quantify Al accumulation across three *Symplocos* species from Central Sulawesi (Indonesia). Earlier observations from literature indicate that out of the 159 specimens of *Symplocos* tested, 158 individuals showed Al accumulation [5,7,15,46,47]. We therefore expect that Al accumulation is a common strategy in *Symplocos*. A second goal is to quantify the intra-tree variation in Al concentration by comparing various organsand plant tissues at different developmental stages. Based on evidence from Al accumulators in the cerrado [48,49] and *Melastoma malabathricum* [24,50], it is expected that the highest levels of Al are found in the leaves and bark tissue, with old leaves showing higher concentrations than younger leaves.

Furthermore, we want to test if Al accumulation in *Symplocos* species shows allelopathic effects on neighbouring species [43] and is associated with potential plant-soil interactions. Considering the high levels of foliar Al in accumulating plants, leaf shedding in the proximity of the stem and subsequent decomposition may have a local effect on the Al concentration and chemical composition in the soil, with potentially higher concentrations of soluble Al under the canopy of an Al accumulating tree than outside its canopy. If this hypothesis would be correct, the high availability of soluble Al could negatively affect the growth and performance of plants with low Al toxicity tolerance growing next to Al accumulators due to increased Al stress. The higher availability of soluble Al should therefore negatively affect the growth and abundance of potentially Al sensitive plants growing next to Al accumulators altering their environment. Therefore, soil samples are analysed at different levels of depth and at different proximities to the stems of *Symplocos* trees.

Finally, we aim to find out if high Al levels are correlated with other elements in aboveground tissues of *Symplocos*. In particular, we expected that Al levels are positively correlated with calcium (Ca) and magnesium (Mg), based on earlier findings of Al accumulating plants [26,42]. These goals are achieved by studying both Al accumulating species and non-accumulating plant species from three study sites in Central Sulawesi.

## Materials and Methods

### Study sites and species

Fieldwork was conducted in October and November 2013 at three different sites in the Lore Lindu National Park in Central Sulawesi. The sites were selected based on the relatively high abundance of *Symplocos* trees based on earlier observations [51], and represented primary montane rainforests with minor differences in altitude and climate (see Table 1 for details). For each site, a single species of *Symplocos* was sampled (Table 1).

**Table 1:** Field sites of montane rainforest selected in Central Sulawesi (Indonesia) with reference to the species studied, geographical information and climate^a^.

Site	Dali	Nokilalaki	Rorekautimbu
Species sampled	*Symplocos odoratissima* Choisy ex Zoll., *Lithocarpus sp*.	*Symplocos ophirensis* C.B.Clarke, *Polyosma celebica* Schulze-Menz	*Symplocos ambangensis* Noot., *Syzygium sp*.
Mean annual precipitation	2,045 mm	1,951 mm	2,131 mm
Mean annual temperature	17.5°C	16.8°C	14.1°C
Latitude Longitude	1°41'34.50"S, 120° 9'23.52"E	1°14'39.00"S, 120° 9'14.00"E	1°16'42.10"S, 120°18'34.30"E
Altitude	1,748 m	1,876 m	2,388 m

^a^Abiotic factors were extracted from the WorldClim database (data for current conditions ~1950–2000).

*Symplocos* trees were represented as understorey plants, about 8 to 15 m tall with a diameter at breast height (DBH) from 5.4 to 25.7 cm. The vegetation showed a high abundance of rattan species (Arecaceae), Rubiaceae, Zingiberaceae and Melastomataceae, and the most prominent tree species were Fagaceae, Myrtaceae and Podocarpaceae. Besides *Symplocos*, a second plant species was sampled at each field-site, including *Lithocarpus sp*. (Fagaceae), *Polyosma celebica* Schulze-Menz (Escalloniaceae), and *Syzygium sp*. (Myrtaceae). The main criterion for selecting these species was their abundance at each field site. Moreover, these additional species represented one additional Al accumulating species (*Polyosma celebica*; [5,52]) and two Al-excluding species (*Syzygium* sp. and *Lithocarpus* sp.).

The soil at the three field sites was composed of undulating red to yellow podsoil with some areas showing alluvium and organosols.

### Collection of plant material

Leaf samples were divided into three developmental stages (i.e., young, mature, and old), which were grouped based on their size and colour. Data on the leaf life span were not available. However, abscission was assumed to provide an additional and easy criterion for distinguishing the fully developed, mature leaves from old ones. Young (i.e., not fully developed) leaves had a light green colour compared to the larger, dark green, mature and fully developed leaves. The old leaves were typically yellowish green. Young and mature leaves were collected from branches. The old leaves were sampled by shaking a tree and picking up the fallen leaves. We also collected old leaves that could be found on the forest floor and did not show any signs of decomposition.

For each specimen, we collected five bulk samples. As for the leaves, one sample included three to ten leaves, depending on the leaf size. We aimed to collect ca. 15 leaf samples per tree. However, it was not always possible to collect five bulk samples of old leaves. Bark samples were taken from the trunk and branches, with five bulk samples per tree sampled (n = ca. 10 per tree). Wood samples were taken from branches only (n = 1 per tree). For the non-*Symplocos* species (Table 1), we collected bulk samples of mature leaves (n = 5 per tree), bark from the main trunk (n = 5), and one root sample per tree.

In order to measure the soil pH and the amount of soluble cations, soil samples were taken at three different depth levels (level 1 = 5–7 cm; level 2 = 30–32 cm; level 3 = 40–42 cm) and at three different distances from the trunk of a *Symplocos* tree (near = 0.15–0.45 m; middle = 0.50–2.25 m; far = 1.85–5.00 m). These places corresponded to the soil area close to the trunk of the tree sampled, half-way the radius of the tree crown, and outside the crown. The three arbitrary distances from the trunk thus depended on the crown width of the tree sampled. Each soil sample had a volume of 0.1 L and was taken with a standardized metallic cylinder. Therefore, we sampled nine soil samples per tree. Each time, we collected two volumes of soil samples: one for pH measurements, and one for elemental analyses. Root samples were taken after obtaining the soil samples from the first excavation hole, which was closest to the tree trunk, by following the main roots until fine root material was visible (n = 1 per tree).

Overall, we collected a total of 270 soil, 290 leaf, 224 bark, 30 root, and 14 wood samples. All samples were stored in plastic bags at ambient temperature for a maximum of five days before further treatments could be applied in the lab. At Tadulako University, leaves were washed with 3% (v/v) HCl, tap-water, and deionized water to clean the leaves and to avoid any potential contamination. Root material for chemical analysis was thoroughly washed with tap water and rinsed with deionized water to avoid soil contamination. All plant organs and tissues were then transferred into paper bags and oven-dried (OP150, LTE, United Kingdom) at 60°C for 48 hours. Soil samples for pH-measurements were air-dried for 48 hours. All samples were then shipped to Ulm University for further chemical analysis, covered by a Material Transfer Agreement. The research and collecting permit was granted by the National Ministry for Science and Technology of Indonesia (RISTEK) in Jakarta under the number 4306/FRP/SM/VIII/2013.

### Soil pH measurements

We mixed 10 g of soil material with 25 mL of deionized water and homogenized the solution for 1 h. After sedimentation, the pH_H_2_O_ (referred to as pH in this paper) was measured three times with a pH-meter (330i, WTW, Germany). Then, the suspension was filtered with a 595 filter paper (Schleicher & Schuell, Dassel, Germany) using a vacuum pump. The filtrates were stored in a fridge at 6°C prior to elemental analysis.

### Elemental analysis

Leaf, bark, root, and wood samples were ground with a centrifuge mill (ZM1, Retsch, Germany). Root samples were not separated into wood and bark tissue for all species. We transferred 50 mg of the ground tissue into a PTFE-vessel before adding 2 mL of concentrated HNO_3_ and 0.5 mL of concentrated HCl. Samples were microwave digested (Mars 5 plus, CEM, Germany) at 200°C (1600 W) for 30 min (15 min Ramp time; 15 min Holding time).The digestions were diluted to 50 mL with a 0.25% (w/v) CsNO_3_-solution as ionic buffer. The filtrate of the soil samples was diluted 1:10 with the same ionic buffer for analysis. The concentrations of Al, Fe, Mg, Ca and K were measured using microwave-plasma atomic emission spectrometry (MP-AES, Agilent 4100, Agilent Technologies, Australia). Calibration ranged from 1–60 mg/L, depending on the elemental concentration of the digestions.

### Statistical analyses

Statistical analyses were computed with RStudio (Version 0.98.1091, RStudio Inc., Boston, USA). Significant differences between mean values were represented using OneWay-ANOVA with Tukey´s Honestly Significant Difference (HSD) for normal distribution and Wilcoxon´s rank sum test for non-normally distributed data. Correlations were calculated with a Spearman´s rank correlation coefficient. Unless mentioned otherwise, the significance level was P < 0.05. The entire dataset including all measurements was provided in the Supporting Information (S1 Dataset).

## Results

### Interspecific variation of Al concentrations

Al concentrations for the three species of *Symplocos* and *Polyosma celebica* were far above 1,000 mg·kg^-1^ dry weight in all tissues and organs measured. Al levels in *P*. *celebica* varied from 6,250 (± 1,487) mg·kg^-1^ (mean value ± SD) to 7,535 (± 1,528) mg·kg^-1^ in bark and leaf tissue, respectively (Table 2). Al levels in *Symplocos* were up to four times higher, ranging from 16,719 (± 1,120) mg·kg^-1^ to 23,383 (± 9,593) mg·kg^-1^ in leaves of *S*. *ambangensis* and *S*. *odoratissima*, respectively, while bark tissue included slightly lower levels of Al, from 7,034 (± 1,978) mg·kg^-1^ in *S*. *odoratissima* to 20,257 (± 5,305) mg·kg^-1^ in *S*. *ambangensis*. The highest Al concentration measured was 49,100 mg·kg^-1^ in old leaves of *S*. *odoratissima*.

**Table 2:** Summary of the elemental concentrations (mean value ± SD) of various plant tissues from three *Symplocos* species and dominant neighbouring plant species collected at tropical montane forest sites in Central Sulawesi.

Tissue^a^	Species	Al strategy	Al [mg·kg^-1^]	Ca [mg·kg^-1^]	Fe [mg·kg^-1^]	Mg [mg·kg^-1^]	K [mg·kg^-1^]
Root	*Symplocos odoratissima*	accumulator	3,244 ± 1,568	7,532 ± 2,953	738 ± 441	2,022 ± 503	5,182 ± 1,046
	*Symplocos ophirensis*	accumulator	11,380 ± 4,060	5,870 ± 7,308	6,394 ± 2,146	3,806 ± 1,731	7,086 ± 5,618
	*Symplocos ambangensis*	accumulator	11,164 ± 4,983	2,596 ± 837	3,148 ± 4,096	1,302 ± 344	5,810 ± 1,776
	*Polyosma celebica*	accumulator	10,046 ± 2,818	1,734 ± 1,026	8,920 ± 7,585	3,748 ± 2,402	3,414 ± 1,367
	*Lithocarpus sp*.	excluder	3,646 ± 1,826	7,230 ± 2,443	1,750 ± 1,133	2,444 ± 651	4,844 ± 1,387
	*Syzygium sp*.	excluder	986 ± 677	3,828 ± 1,045	276 ± 136	1,264 ± 141	3,176 ± 450
Bark	*Symplocos odoratissima*	accumulator	7,034 ± 1,978	23,939 ± 5,894	13 ± 18	1,169 ± 569	8,194 ± 1,683
	*Symplocos ophirensis*	accumulator	24,400 ± 3,179	15,689 ± 4,629	65 ± 184	2,033 ± 811	9,949 ± 3,144
	*Symplocos ambangensis*	accumulator	20,257 ± 5,305	8,312 ± 3,215	36 ± 61	1,442 ± 1,044	8,097 ± 2,870
	*Polyosma celebica*	accumulator	6,250 ± 1,487	9,602 ± 2,166	38 ± 47	1,552 ± 239	3,054 ± 992
	*Lithocarpus sp*.	excluder	69 ± 21	15,707 ± 2,549	14 ± 14	1,615 ± 687	3,164 ± 674
	*Syzygium sp*.	excluder	32 ± 72	7,224 ± 2,916	52 ± 62	1,140 ± 527	4,194 ± 1,618
Leaf	*Symplocos odoratissima*	accumulator	23,383 ± 9,593	21,274 ± 8,225	91 ± 136	2,197 ± 985	7,290 ± 3,952
	*Symplocos ophirensis*	accumulator	21,352 ± 2,802	9,288 ± 1639	29 ± 18	6,785 ± 1,209	8,807 ± 3,021
	*Symplocos ambangensis*	accumulator	16,719 ± 1,120	8,182 ± 2,603	31 ± 19	2,808 ± 781	7,513 ± 1,325
	*Polyosma celebica*	accumulator	7,535 ± 1,528	8,826 ± 3,195	24 ± 17	5,560 ± 1,946	10,002 ± 4,241
	*Lithocarpus sp*.	excluder	176 ± 140	4,308 ± 2,827	29 ± 99	1,672 ± 527	5,241 ± 1,825
	*Syzygium sp*.	excluder	34 ± 13	3,761 ± 1,319	15 ± 14	1,730 ± 300	10,132 ± 1,995

^a^ Bark tissue included both samples from the trunk and branches, leaf material included young, mature and old leaves.

Much lower Al concentrations were reported for the Al excluding species, with values ranging from 34 (± 13) mg·kg^-1^ (mean ± SD) in leaf tissue of *Syzygium* sp. to 176 (± 140) mg·kg^-1^ in leaves of *Lithocarpus* sp. At the root level, Al concentrations between Al excluders and accumulators were less variable: *Lithocarpus* sp. showed similar mean Al concentrations in its roots than *Symplocos odoratissima*. Also, *Syzygium* showed a relatively high mean Al concentration of 986 (± 677) mg·kg^-1^ in its roots.

### Intra-tree variation of Al accumulation in *Symplocos*

**Fig 1:**
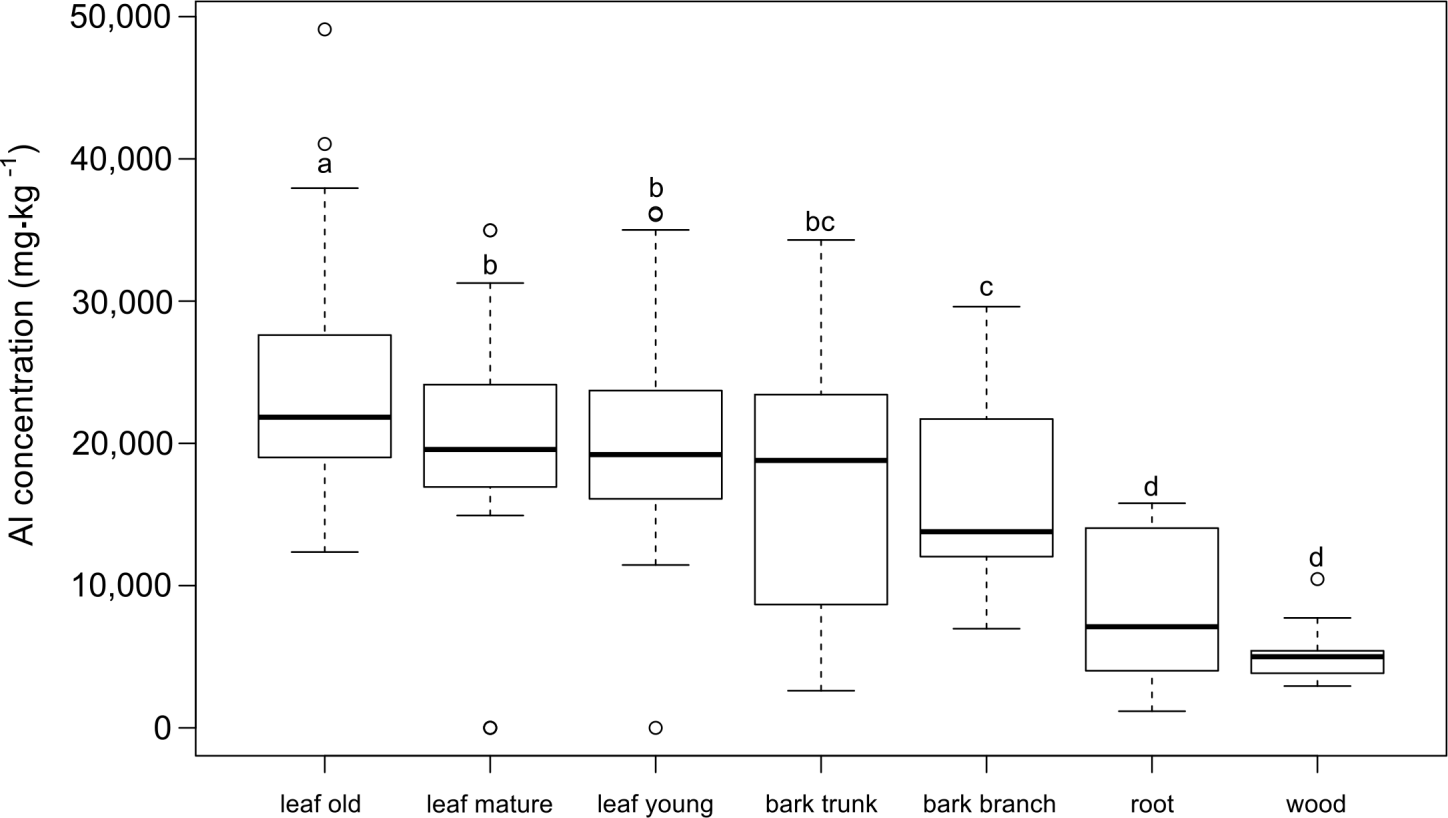
Aluminium (Al) concentrations in various plant tissues for three *Symplocos* species from Central Sulawesi (mg·kg^-1^ dry mass). For each species, five individual trees were measured, with ca. five large samples per tree for leaf and bark material, and one root and wood sample per tree. Leaves were classified according to three developmental stages (young, mature, and old). Different letters indicate significant differences between mean values (Wilcoxon Rank Sum Test, P < 0.05). Boxes show the median, 25th and 75th percentiles, error bars show 10th and 90th percentiles, and individual points show outliers.

We found significant differences in Al concentration between different organs and plant tissues for the three *Symplocos* species studied (Fig 1). The lowest Al concentrations were measured in wood samples (on average 5,181 ± 2,032 mg·kg^-1^) and the highest values in old leaves (24,180 ± 7,236 mg·kg^-1^; S1 Table). Although the roots had a higher mean Al concentration (11,272 ± 4,792 mg·kg^-1^) than the wood, no significant difference was found between these organs. There was also no significant difference between bark tissue from the main trunk and small branches (Fig 1). Al levels in the bark of the main trunk were similar to those in young and mature leaves (Fig 1). Therefore, young and mature leaves did not show a significant difference (P = 0.615, Fig 1), but a significantly higher Al concentration was found for the old leaves compared to younger leaf ages for all three species (P < 0.01, Fig 1).

**Fig 2:**
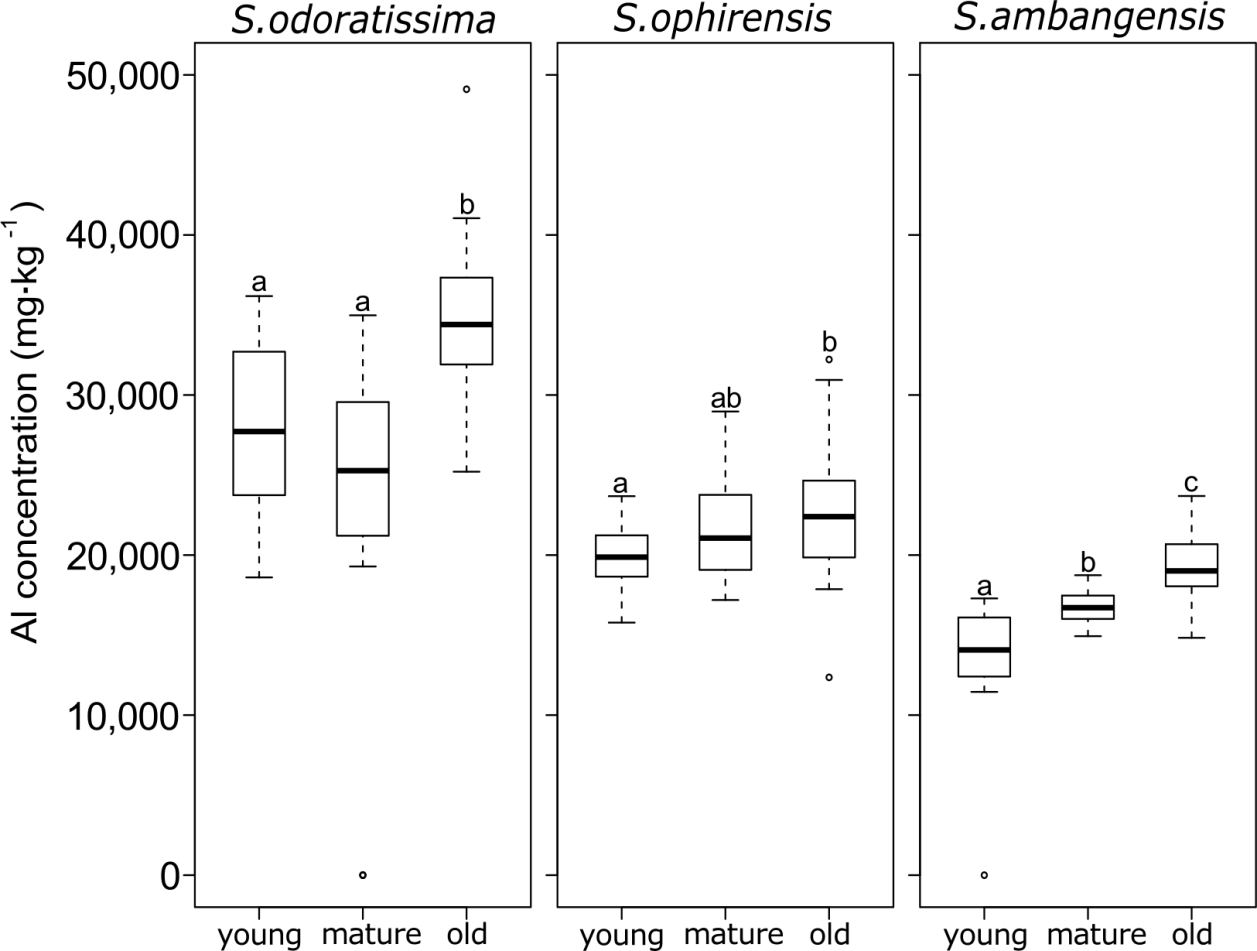
Boxplots showing aluminium (Al) concentrations (mg·kg^-1^ dry mass) in leaves for three species of *Symplocos*, showing the median, 25th and 75th percentiles, 10th and 90th percentiles as error bars, and outliers as individual points. Leaves were grouped into three different developmental stages (young, mature, and old). Different letters indicate significant differences between mean values (Wilcoxon Rank-Sum Test, P < 0.05).

When analysing the three *Symplocos* species separately, no significant difference between mature and young leaves could be found for *S*. *odoratissima* and *S*. *ophirensis* (Fig 2), but significant differences between the three developmental leaf stages were found for *S*. *ambangensis*. For the three *Symplocos* species studied, old leaves showed significantly higher Al concentrations than younger developmental leaf stages.

### Soil characteristics

**Fig 3:**
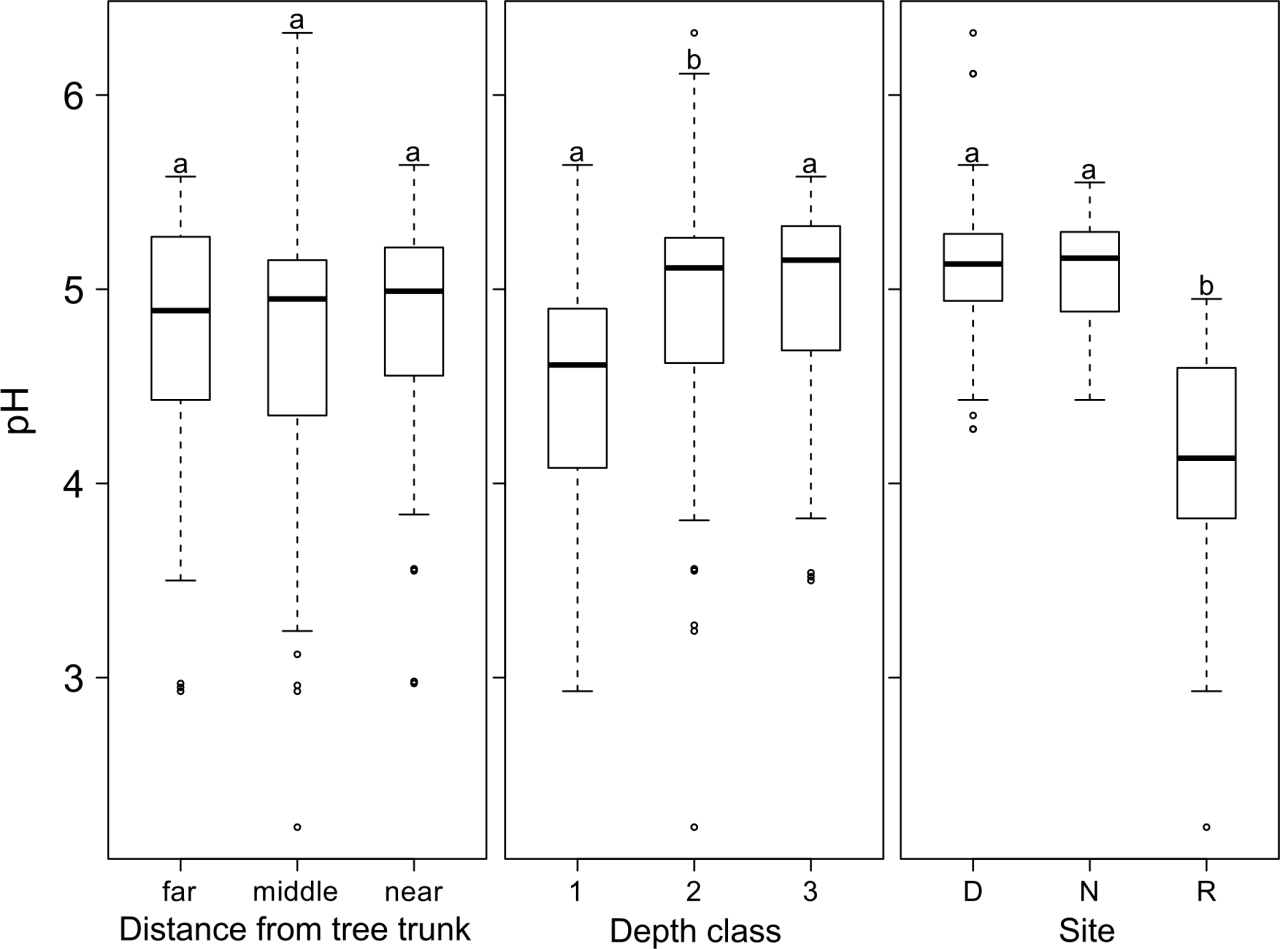
pH_H_2_O_ values of soil samples (n = 135) from the surface soil layer of *Symplocos* trees in Central Sulawesi. Five individual trees per species were sampled for three species at three field sites (D = Dali, N = Nokilalaki, R = Rorekautimbu). The pH values are shown in relation to 1) the distance from the tree trunk, 2) the soil depth, and 3) the sampling site. Distances from the tree trunk depended on the tree crown width (near = at 0.15 to 0.45 m from the trunk; middle = at 0.50 to 0.25 m from the trunk, i.e. half the radius of the tree crown; far = 1.85–5.00 m, i.e. outside the tree crown). Depth classes 1, 2, and 3 were at 5 to 7 cm, 30 to 32 cm, and 40 to 42 cm, respectively. The box plots show the median, 25th and 75th percentiles, error bars show 10th and 90th percentiles, and individual points show outliers. Different letters indicate significant differences between mean values (Wilcoxon Rank-Sum Test, P < 0.05).

The soil pH values measured varied from 2.2 to 6.3. With an average value of 4.7 (± 0.61) the soil at the three sites was acidic. Furthermore, the mean soil pH at the Rorekautimbu site was 4.1 (± 0.52), which was significantly lower than in Dali and Nokilalaki (P < 0.001). The pH values showed a homogeneous pattern, regardless of the relative distance between the main trunk and the tree crown (Fig 3). However, at all sites we found a significantly lower pH-value in the top-soil layer compared to deeper soil layers (P < 0.001 Fig 3). No correlation was found between the amount of soluble soil ions (Ca, K, Mg, Fe) and the pH values measured (Table 3).

**Table 3:** Elemental concentration of water extracted soluble ions^a^ in soil samples from three montane rainforest field sites in central Sulawesi. The values represent mean values (± SD) for three depth classes near five *Symplocos* trees sampled per site. For each tree, nine soil samples were taken.

Element	Depth^b^	Dali [mg·kg^-1^]	Nokilalaki [mg·kg^-1^]	Rorekautimbu [mg·kg^-1^]
Al	1	27 ± 20	38 ± 36	235 ± 290
	2	30 ± 43	25 ± 38	84 ± 103
	3	36 ± 79	5 ± 7	139 ± 281
Ca	1	140 ± 222	28 ± 21	28 ± 33
	2	52 ± 61	10 ± 7	26 ± 70
	3	33 ± 43	6 ± 7	18 ± 23
Mg	1	58 ± 35	143 ± 118	73 ± 62
	2	22 ± 16	54 ± 39	23 ± 31
	3	19 ± 16	19 ± 15	19 ± 24
Fe	1	6 ± 4	823 ± 1426	383 ± 498
	2	9 ± 14	323 ± 720	103 ± 185
	3	11 ± 22	6 ± 18	202 ± 532
K	1	309 ± 142	313 ± 86	327 ± 150
	2	209 ± 83	73 ± 46	103 ± 100
	3	190 ± 80	26 ± 18	85 ± 59

^a^ 10 g of soil suspended in 25 mL of demineralised water.

^b^ Depth classes: 1 = 5–7 cm, 2 = 30–32 cm, and 3 = 40–42 cm.

In general, the uppermost layer had a higher concentration of soluble ions, compared to the middle and bottom layer. The highest concentration of soluble Al was found in the soil layers at the site in Rorekautimbu with 235.16 (± 289.53) mg·kg^-1^ in the uppermost soil layer (Table 3), while soluble Al concentrations did not exceed 50 mg·kg^-1^ at the two other field sites. Ca concentrations were highest in Dali with 139.83 (± 220.01) mg·kg^-1^and low in Nokilalaki and Rorekautimbu. The concentration of soluble Fe was higher in Nokilalaki (822.66 ± 1426.07 mg·kg^-1^) and Rorekautimbu (383.33 ± 497.90 mg·kg^-1^) than in Dali (6.16 ± 4.41 mg·kg^-1^).

The soil elemental concentrations did not differ significantly along the three distances of the tree crown (data not shown). No correlation between the soil soluble elements was found.

### Al concentrations in relation to other elements

**Fig 4:**
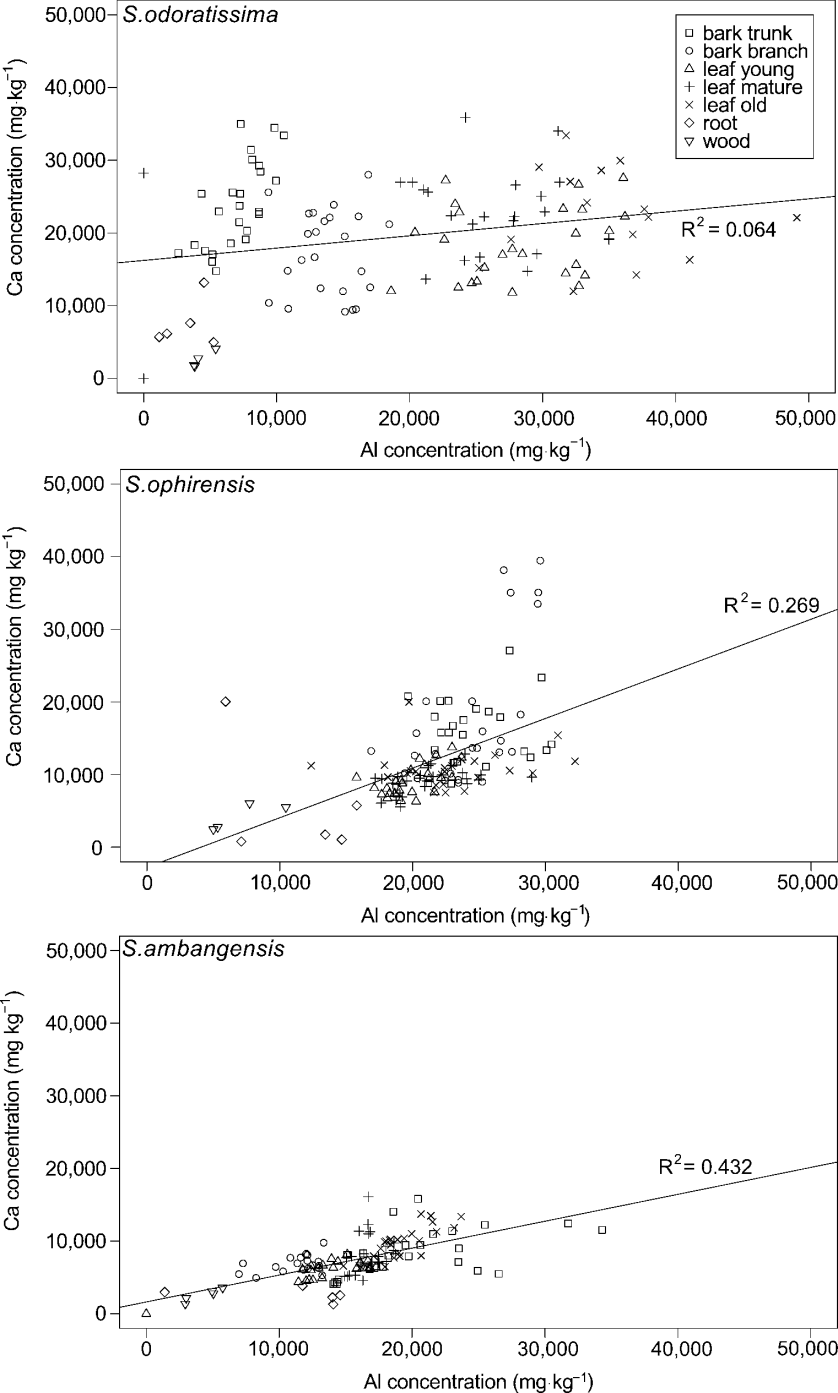
Correlation between aluminium (Al) and calcium (Ca) concentrations in three *Symplocos* species from Central Sulawesi. Five trees were sampled for each species. Different symbols represent different plant tissues and organs. The solid line represents the regression line. *S*. *odoratissima*: P = 0.02, n = 124; *S*. *ophirensis*: P < 0.01, n = 134; *S*. *ambangensis*: P < 0.01, n = 135.

No correlation was found between Al and the other elements measured, including Fe, Mg and K. The Al concentration in the three *Symplocos* species was positively correlated with the concentration of calcium (Ca), both at the leaf level and the entire plant level (Fig 4). At the whole plant level, the correlation was significant for *S*. *odoratissima* (R^2^ = 0.064, P = 0.02), *S*. *ophirensis* (R^2^ = 0.269, P < 0.01), and *S*. *ambangensis* (R^2^ = 0.432, P < 0.01). At the leaf level the Al-Ca correlation was strongest for *S*. *ambangensis* (R^2^ = 0.537, P < 0.01), significant for *S*. *ophirensis* (R^2^ = 0.133; P < 0.01), but weak and non-significant (R^2^ = 0.087; P = 0.33) for *S*. *odoratissima* (data not shown). The leaf Ca concentration was significantly lower in both *Syzygium* sp. and *Lithocarpus* sp. compared to the *Symplocos* species. Leaf Ca concentration in *P*. *celebica* similar as the mean Ca levels in *S*. *ophirensis* and *S*. *ambangensis*.

Leaf age had a considerable effect on Ca concentration, with the concentration in the young leaves significantly lower than in old leaves (P = 0.0078, data not shown). However, there was no significant difference in the amount of Ca between young and mature, and between mature and old leaves for the four Al accumulating species. There is a significant, age dependent increase in the Ca concentration for *S*. *ophirensis* and *S*. *ambangensis*, but not for *S*. *odoratissima* (data not shown).

No correlation was found between Al and the other elements measured, including Fe, Mg and K.

## Discussion

### Al accumulators and excluders

Our chemical analyses clearly indicate that the genus *Symplocos* includes strong Al-accumulating species in Indonesia. Therefore, our data support the old, pre-Linnean name given by Rumphius (*Arbor aluminosa*) to these Al accumulating trees, although the values measured are not as high as the 72,240 mg·kg^-1^ recorded for *S*. *spicata* [4]. Mean Al levels measured in *S*. *crassipes* from Brunei included 33,883 mg·kg^-1^ [15], which is slightly higher than the values obtained for *S*. *odoratissima*. Temperate to subtropical species of *Symplocos*, however, appear to show slightly lower levels of Al accumulation, with a mean value of 8,872 mg·kg^-1^ for five Japanese species [14] and an average concentration of 8,309 (± 282) mg·kg^-1^ for *S*. *chinensis* grown in a standard nutrient solution with 0.5 mmol·L^-1^ Al [16]. Differences in soil acidity and/or leaf longevity could explain the lower Al levels in temperate species of *Symplocos* compared to tropical species. Al values of more than 10,000 mg·kg^-1^ were also reported for tea (*Camellia sinensis*) plants growing in their natural environment [53], which supports the close phylogenetic relationship between Symplocaceae and Theaceae.

*Lithocarpus* and *Syzygium* show Al concentrations that are characteristic of Al excluders growing in tropical, acidic soils, while Al accumulation is supported for *Polyosma* [5]. With casparian stripes blocking apoplastic transport around the central, vascular bundle in roots [54], it can be suggested that symplastic pathways between Al accumulators and excluders are likely different, probably through channels that either promote or block the uptake of Al [34]. The relatively low Al concentration in root samples of *Syzygium* (986 ± 677 mg·kg^-1^) seems to suggest that this genus may have a mechanism that prevents Al uptake into its root tissue. Representatives of the Myrtaceae family (*Melaleuca leucadendra*, *Melaleuca cajuputi* and *Eucalyptus camaldulensis*) that were exposed to Al (0.5 mmol to 4 mmol) showed various Al tolerance mechanisms (e.g., alteration of the pH at the root level) in addition to the exudation of organic compounds and phosphate to alleviate Al toxicity [55]. Besides the genera *Eugenia* and *Xanthostemon*, Al accumulation has not been reported in Myrtaceae [56]. However, the physiological explanation for the relatively low concentration of Al in the root tissue of *Syzygium* requires further research.

### Al concentrations between various tissues of *Symplocos*

Earlier findings suggest that the xylem tissue represents the major transport pathway for Al between the roots and the leaves of Al accumulating tea plants [53]. Therefore, the concentration of Al can be expected to increase in leaves by the transpiration stream. Since the old leaves were found to show the highest Al concentrations, immobility of Al within the leaf tissue can be suggested and is in line with earlier findings of Al storage in leaf epidermal tissue and xylem [16,24,38]. The evidence that old leaves of *Symplocos* show the highest Al levels is important for the traditional weavers in Indonesia because a lower amount of *Symplocos* material will be required to obtain a similar dye efficiency for a given amount of textile. Moreover, harvesting of old leaves from the forest floor is unlikely to result in tree death, unlike cutting aboveground stems and trunks.

High aluminium concentrations in the bark may also suggest transport via the phloem, which was found for instance in *Camellia oleifera and C*. *sinensis* [57,58]. It is unclear, however, if transport via the phloem mainly follows an axial direction via the phloem sieve tubes, or whether Al shows possible interactions between xylem and phloem tissues, with both axial and radial transport via ray cells. Regardless of the transport pathways, we found high levels of Al in the ray parenchyma and phloem of the plants sampled in Indonesia (M.S., unpublished data) based on Al specific staining techniques.

### The elemental allelopathy hypothesis and soil properties

As expected, the highest concentrations of soluble cations are found in the top layer of the soil, and can be explained by decomposition of organic matter, which makes various nutrients available for recycling [7,59]. Furthermore, the degradation of organic material lowers the pH through bacterial synthesis of carbonic acids in the top layer. This also promotes the availability of soluble cations through exchange of protons in the substrate [60].

The soil pH and the soluble nutrients did not show any correlation. Thus, a lower pH does not account for a degradation of substrates to make nutrients soluble, but will shift their chemical forms (i.e., a shift from aluminium hydroxide forms to Al^3+^) [19,30,61].

The elemental allelopathy hypothesis for Al could not be confirmed because no difference in the amount of soluble Al in the soil and in the soil pH could be found between the different proximities to the stem. Active acidification through release of protons at the root zone level was not tested. Even if such an acidification mechanism would be applied by *Symplocos* trees to facilitate Al accumulation, it could be expected that a low pH value may occur in the proximity of the stem [24]. Because our pH values were measured at a relatively large (macroscopic) scale, it is possible that pH gradients may occur at a local scale close to the fine roots, which could then account for a higher solubility of Al species in the soil.

Interestingly, Al accumulating representatives of various families (e.g., Melastomataceae, Rubiaceae, Theaceae, Escalloniaceae) were growing in close proximity to members of Al excluding families (e.g., Zingiberaceae, Arecaceae, Myrtaceae, Moraceae). Although we did not conduct any detailed measurements on growth rate, reproductive success, vegetation density, life span, etc., Al accumulators and excluders appear not to show any obvious ecological advantage or benefit for dealing with Al toxicity in acidic soils.

Our observations of vertebrate and/or invertebrate herbivory in the *Symplocos* species studied suggest that Al accumulation does not result in an enhanced defence mechanism. However, it was not clear whether the herbivores attacking the *Symplocos* leaves were specialists or generalists, and whether Al accumulation provides higher resistance to pathogens than Al excluding plants. Sika deer in Japan were reported to avoid bark stripping for *Symplocos coreana* and neotropical leaf cutting ants were shown to have a low appetite for leaves of the Al accumulating *Coussarea hydrangeifolia* (Rubiaceae) [62,63]. Observation of leaves from our three *Symplocos* species frequently showed signs of herbivory (S1 Fig).

Al accumulation in plants differs considerably from accumulators of transition metals or arsenic (As) [64–67]. For instance, Al accumulation frequently characterises large taxonomic groups at the genus and (sub)family level, unlike heavy metal accumulators [13,14]. Therefore, it can be suggested that Al accumulation conveys a selective advantage in a wider range of ecological contexts than heavy metal accumulators. While heavy metals are restricted to metalliferous soils and to active or abandoned mining sites, Al is omnipresent in all soil types, but the amount of soluble Al strongly depends on soil pH [68]. As far as we know, distinct soil patches that represent “geochemical islands” with higher levels of available aluminium have only been investigated for the Al tolerant species *Plantago almogravensis* [69]. The distribution density of this species was linked with the realized niche concept [70] and high Al tolerance was found to provide a successful strategy for *P*. *almogravensis* to grow on acidic and Al rich spots whereas it would be outcompeted on habitats with reduced Al toxicity. To what extent Al exclusion and accumulation may have an effect on tropical forest dynamics remains unclear and would require detailed observations at the population level [71,72].

### Correlation between Al and other elements

Compared to data from Masunaga et al. [42], the Ca values for leaves and bark of Al accumulators are rather low, especially in *Polyosma celebica*. Based on a global dataset on foliar elemental concentrations, the values for Mg, K and Ca concentrations in *Symplocos* are similar to our data [14,15].

As expected, our data show that Ca levels are correlated with Al, both at the whole plant level, and to a lesser degree at the leaf level (Fig 4). This finding is strengthened by the lack of a correlation of soluble ions in the soil and is thus only found in the plant body. The positive correlation between Ca and Al confirms previous studies that showed an increase in Ca uptake with enhanced Al levels [42,73], and a Ca-Al correlation was also found in the sister family Theaceae [74], which may provide evidence for a genetic disposition of the mechanisms underlying the uptake of Ca in the presence of Al. At the leaf level, there is a clear difference between the excluding species and the accumulators. The genus *Symplocos* is known to have calcium oxalate crystals stored in its leaf tissue [16,75]. This could contribute to the higher foliar concentrations in *Symplocos* when compared to the neighbouring plants, at least in case of *Syzygium* and *Lithocarpus*. No calcium oxalate crystals were reported for *S*. *ophirensis* [75], which could explain the lower concentrations of foliar Ca when compared to the other two *Symplocos* species studied. However, as the correlation at the leaf level is not as strong as at the whole-plant level, these crystals cannot be the only explanation. Oxalate can bind to both Ca and Al and could therefore act as a regulating molecule for the accumulation of both elements. The formation of crystals with metal inclusion and subsequent storage in the vacuole is also hypothesized to be a possible mechanism to reduce the toxic effect of Al and other metals [76]. The correlation between Al and Ca concentrations also provides evidence for the relative immobility of both elements once they are taken up by leaf tissue, where Ca is known for its immobilization from old to young leaves, even under stressful conditions [77].

The lower concentration of Fe in the root tissue of *S*. *odoratissima* could be explained by low soluble Fe concentrations in the soil (Table 3), whereas both *S*. *ophirensis* and *P*. *celebica* showed higher root Fe concentrations because of higher Fe levels in the soil. Adsorption at the root level does not affect the potential uptake into the aboveground tissues. Furthermore, our experimental setup did not account for desorption of adsorbed metals in the root tissue, which is why the root elemental concentrations are not useful to discuss uptake in the aboveground plant tissues. However, any given difference between accumulators and excluders could still suggest the potential exudation of chelating agents that may affect Al uptake into the root tissue and subsequently uptake into aboveground tissues [78]. Whether both Fe and Al are bound to the symplast or apoplast was not addressed in this study.

### Conclusion

Our finding that the highest Al levels occur in the oldest leaves should provide important information for Indonesian weavers to achieve sustainable harvesting of *Symplocos* plant material for their traditional dying techniques [12]. The results indicate that there are transport pathways for Al within the different tissues of Al accumulators, whereas non-accumulators seem to possess mechanisms that actively block the uptake of Al at the root level. No ecological advantage or disadvantage was found with respect to the elemental allelopathy hypothesis in the case of Al. Al excluders were growing in close proximity to accumulating species, suggesting that both accumulation and exclusion seem to be equally successful strategies to deal with Al toxicity. Furthermore, calcium is suggested to play a role in Al accumulation of *Symplocos* species.

## Supporting Information

S1 Dataset The dataset including all elemental concentrations measured with microwave-plasm atomic emission spectrometry (MP-AES) and soil pH values at three montane rainforest sites in Central Sulawesi. (XLSX)

S1 Fig Leaves of *Symplocos odoratissima* showing signs of herbivory in a montane rainforest at the Dali field site in Central Sulawesi. (TIFF)

S1 Table Elemental concentrations of different organs and developmental stages of leaves for three *Symplocos* species from three montane rainforest sites in Central Sulawesi. (DOCX)
