# Supplementary figures and images for: Aluminium Accumulation and Intra-Tree Distribution Patterns in Three Arbor aluminosa (Symplocos) Species from Central Sulawesi
Source: PLoS One. 2016 Feb 12;11(2):e0149078. doi: 10.1371/journal.pone.0149078 (PMC4752314; doi:10.1371/journal.pone.0149078)

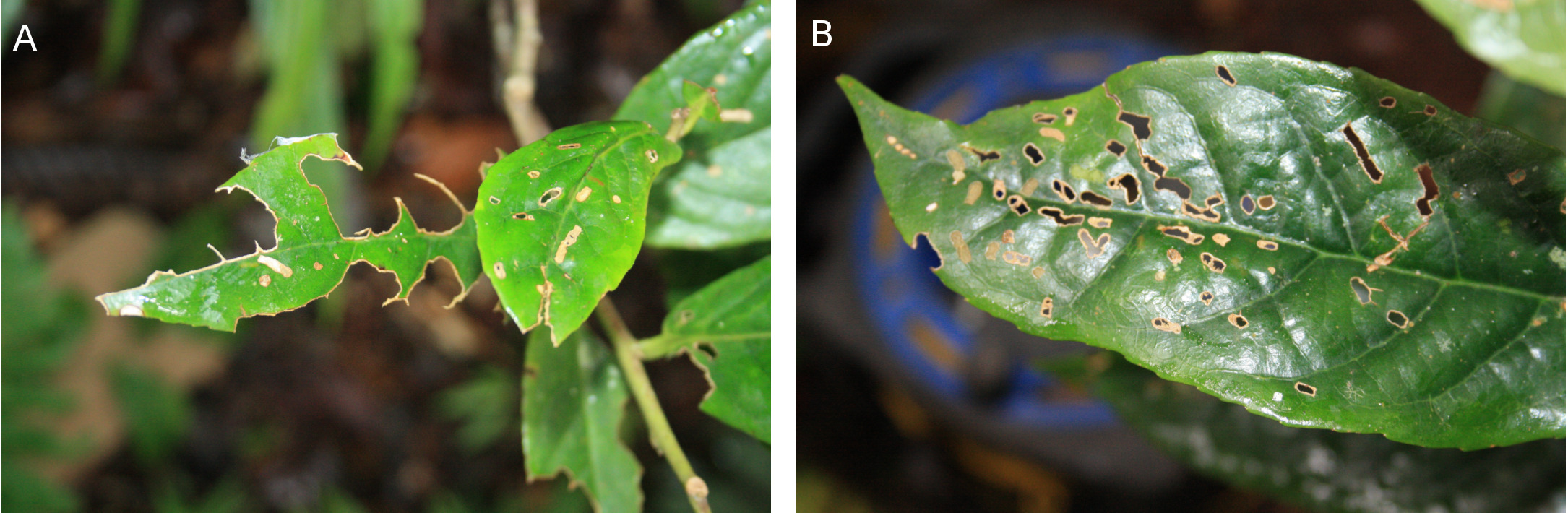

Supplement: S1 Fig — (TIFF) [file pone.0149078.s002.tiff]
