# Supplementary material for: Aluminium Accumulation and Intra-Tree Distribution Patterns in Three Arbor aluminosa (Symplocos) Species from Central Sulawesi
Source: PLoS One. 2016 Feb 12;11(2):e0149078. doi: 10.1371/journal.pone.0149078 (PMC4752314; doi:10.1371/journal.pone.0149078)
Supplement: S1 Table — (DOCX) [file pone.0149078.s003.docx]

**S1 Table**. Elemental concentrations of different organs and developmental stages of leaves for three *Symplocos* species from three montane rainforest sites in Central Sulawesi.

| Tissue | Specification | Al [mg·kg^-1^] | Ca [mg·kg^-1^] | Mg [mg·kg^-1^] | Fe [mg·kg^-1^] | K [mg·kg^-1^] |
| --- | --- | --- | --- | --- | --- | --- |
| Leaf | Old | 24,180 ± 7,236 | 13,049 ± 6,349 | 4,083 ± 2,363 | 62 ± 61 | 3,322 ± 2,167 |
| Leaf | Mature | 20,485 ± 6,484 | 12,915 ± 7,853 | 3,930 ± 2,285 | 51 ± 86 | 7,870 ± 3,067 |
| Leaf | Young | 20,708 ± 7,025 | 11,217 ± 6,269 | 3,757 ± 1,949 | 21 ± 50 | 9,276 ± 3,860 |
| Bark | Trunk | 17,231 ± 8,356 | 15,980 ± 7,985 | 1,548 ± 912 | 38 ± 116 | 8,747 ± 2,795 |
| Bark | Branch | 16,578 ± 6,228 | 13,875 ± 8,356 | 2,012 ± 1,136 | 38 ± 53 | 10,970 ± 3,124 |
| Wood |  | 5,181 ± 2,032 | 3,081 ± 1,411 | 856 ± 500 | 177 ± 255 | 5,158 ± 1,738 |
| Root |  | 8,596 ± 5,566 | 5,332 ± 5,190 | 2,376 ± 1,545 | 3,426 ± 3,668 | 6,026 ± 3,668 |

Concentrations are given as mean values ± SD.

n = 15 trees in total, including five trees per species and three different species.
